# Longitudinal relationships among problematic mobile phone use, bedtime procrastination, sleep quality and depressive symptoms in Chinese college students: a cross-lagged panel analysis

**DOI:** 10.1186/s12888-021-03451-4

**Published:** 2021-09-10

**Authors:** Guanghui Cui, Yongtian Yin, Shaojie Li, Lei Chen, Xinyao Liu, Kaixuan Tang, Yawen Li

**Affiliations:** 1 School of Acupuncture and Tuina, Shandong University of Traditional Chinese Medicine, Jinan, 250355 China; 2 School of Nursing, Shandong University of Traditional Chinese Medicine, Jinan, 250355 China; 3 Department of Social Medicine and Health Service Management, Xiangya School of Public Health, Central South University, Changsha, 410078 China; 4 School of Chinese Medicine, Shandong University of Traditional Chinese Medicine, Jinan, 250355 China; 5 School of Medicine, Shandong University of Traditional Chinese Medicine, Jinan, 250355 China; 6 School of Ophthalmology and Optometry, Shandong University of Traditional Chinese Medicine, Jinan, 250355 China

**Keywords:** Problematic mobile phone use, Bedtime procrastination, Sleep quality, Depressive symptoms, Longitudinal relationship

## Abstract

**Background:**

Cross-sectional and longitudinal studies have found that problematic mobile phone use, bedtime procrastination, sleep quality, and depressive symptoms are strongly associated. However, studies are inconsistent regarding whether problematic mobile phone use predicts depressive symptoms or vice versa, and sleep factors have been infrequently focused on in this regard. In addition, few studies have examined the longitudinal associations and directions of effects between these factors. Therefore, this study aims to explore the longitudinal relationship among problematic mobile phone use, bedtime procrastination, sleep quality, and depressive symptoms in college students.

**Methods:**

Overall, 1181 college students completed questionnaires on problematic mobile phone use, bedtime procrastination, sleep quality, and depressive symptoms at two time points 12 months apart. A cross-lagged model was used to examine the longitudinal relationship between these factors.

**Results:**

Cross-lagged analyses showed significant bidirectional relationships of problematic mobile phone use with bedtime procrastination and depressive symptoms. Additionally, there were also significant bidirectional relationships of sleep quality with bedtime procrastination and depressive symptoms. Problematic mobile phone use predicted subsequent sleep quality one-way, and bedtime procrastination predicted subsequent depressive symptoms one-way.

**Conclusions:**

This study further expands our understanding of the longitudinal and bidirectional relationships among problematic mobile phone use, bedtime procrastination, sleep quality and depressive symptoms and helps school mental health educators design targeted interventions to reduce problematic mobile phone use, sleep problems, and depressive symptoms among college students.

**Supplementary Information:**

The online version contains supplementary material available at 10.1186/s12888-021-03451-4.

## Introduction

With the rapid development of Internet technology, electronic products such as mobile phones have become one of the main tools for individuals to access and supply information [1]; conduct interpersonal communication; obtain entertainment, diversion, and relaxation; receive monetary compensation (such as finding bargains on product and services to save money, getting profitable financial information, or working and doing tasks to make money) [2]; and pursue other activities (such as education and health management), by virtue of their devices’ convenience, accessibility, and powerful functions [3, 4]. However, in the process of using these electronic products and the functions they enable, human beings often experience a variety of problematic behaviors, including overuse and dependence [5], which have gradually become the focus of academic attention. Among groups that typically have access to their own phones, adolescents and young adults, especially college students, are more likely to experience problematic mobile phone use (PMPU) because they have more free time, lower levels of self-control, and increased identity and lifestyle needs (such as online learning, social interaction, games, and shopping) [6–8]. A meta-analysis pointed out that the prevalence of PMPU among Chinese college students was as high as 23% [9]. In a previous study, PMPU was defined as uncontrolled or excessive use of mobile phones by individuals that causes problems in daily life [10]. In some studies, it was also referred to as mobile phone dependence [11], mobile phone addiction [12], and smartphone addiction [13]. Similar to the symptoms of substance use disorder, uncontrolled and excessive use was the important symptoms and characteristics of PMPU [14]. In this concept, uncontrolled use was considered a core feature of PMPU, meaning, although aware of the adverse effects, individuals still used and had difficulty controlling the use of mobile phones [10]. Further, excessive use means that an individual’s mobile phone use exceeds a certain time and range. Obviously, this requires a demarcation point to determine whether an individual has excessive mobile phone use. Based on the existing research, it is not feasible to determine the cut-off point by using quantitative methods such as time and frequency, because the motivations and natures or modes of mobile phone use of different individuals are very heterogeneous [15]. For example, when mobile phones were used to contact families and friends, provide social support, or when learning or working with an aim to increase productivity or self-improvement (such as participating in online work conferences, browsing online learning resources, information retrieval, and schedules) [16], the use time of mobile phone can be long and may not have negative consequences; such situations should not be categorized as excessive mobile phone use [17]. Therefore, on the basis of considering the motivation, nature or mode of mobile phone use, current studies tend to use evaluation from the perspective of others (for example, my classmates say that I use mobile phones for too long and too often), rather than quantitative methods to judge excessive use. Specifically, when evaluating excessive use, it need to declare to participants that the mobile phone usage in the evaluation refers to mobile phone use patterns or content are uncontrolled online games, social media, or entertainment (e.g., watching movies and listening music)—and the reason or motivation for use is evasion of reality, failure to regulate stress and negative emotions, and boredom, fear of missing out [14] or specific personality traits such as shyness [10]. In addition, PMPU also showed two characteristics: tolerance (the frequency and duration of mobile phone use by individuals to achieve satisfaction have increased significantly) and withdrawal (individuals experience psychological withdrawal symptoms such as panic, restlessness, and irritability when separated from their mobile phone) [18]. Therefore, in this study, we define PMPU as an individual’s uncontrolled or excessive use of mobile phones and adverse effects when performing activities with the motivation and purpose of relieving negative emotions, relaxing oneself, and satisfying online social and entertainment needs, rather than activities with the motivation and purpose of self-improvement, increase productivity or search for social support, such as work, study, and communication with families and friends.

The concept of PMPU is somewhat controversial, because the main function of mobile phones was the operation of Internet-based applications [19], which indicates that PMPU has many similarities with Internet-based addictions, such as gaming disorders, and may have mutual influences. For example, individuals with Internet addiction are more likely to experience PMPU, and vice versa. Previous research has shown that there was a positive correlation between Internet addiction and PMPU [20]. However, studies have also shown differences between the two in risk factors such as gender and personality characteristics [21]. For example, men experience more Internet addiction, while women demonstrate more PMPU [22]. However, it should be noted that mobile phones not only provide functions such as the Internet and games, but also have various other services and functions such as communications, cameras, multimedia playback, painting, and e-book reading. These services may not be related to the Internet. In addition, individual Internet use relies not only on mobile phones but also on desktop laptops, computers, or digital tablets. Therefore, some symptoms of PMPU may be different from those of Internet addiction. In addition, a study found that, compared with using their phones for playing games, individuals with PMPU were more likely to use social networks [23], which indicates that there may also be differences between PMPU and gaming disorders. Therefore, current research tends to treat PMPU as an independent concept, and has developed some specific assessment tools [15, 24–26], which are widely accepted and recognized by scholars. However, it should be noted that none of the existing scales can fully consider the characteristics of uncontrolled or excessive, tolerance and withdrawal to evaluate PMPU [15]. Thus, this study used the Mobile Phone Addiction Tendency Scale (MPATS), which is widely used in mainland China, to focus on the evaluation of uncontrolled use, excessive use evaluated by classmates or friends, tolerance, withdrawal symptoms, and negative consequences [27].

### PMPU and depressive symptoms

Previous research has shown PMPU to be associated with individual health status and to not only cause physical symptoms such as musculoskeletal pain [28] and increase the risk of traffic injuries [29] but also induce mental health problems [30]. Regarding physical and mental health conditions related to PMPU, depressive symptoms have been widely investigated by scholars. A meta-analysis of 33,650 college students in 40 studies showed that PMPU was significantly positively correlated with depression [31]. In addition to exploring the direct relationship per se, an important research question is the nature of the potential mechanism between PMPU and depressive symptoms. There have been many studies looking at mediators of the relationship (from self-esteem [32] and self-determination [33] to personality traits such as mindfulness [34], attachment variables [35], interpersonal relationships [36], and stress and burnout [5], among others). However, most of these studies adopt cross-sectional designs, failing to demonstrate the directions of effects between various variables, which makes it difficult to truly understand the associations among PMPU, depressive symptoms, and other potential influencing/mediating factors. It seems premature to investigate possible mechanisms of a relationship of which both the direction and causality have not been properly established. Therefore, before paying attention to the mechanism of this relationship, longitudinal research is urgently needed to determine its direction. Existing studies have focused on the longitudinal relationship between PMPU and depressive symptoms, but their results have been contradictory (i.e., the directionality is inconsistent). For example, a three-year follow-up study of 1877 Korean adolescents using autoregressive cross-lagged model analysis found a bidirectional longitudinal relationship between PMPU and depressive symptoms [37]; however, a longitudinal study in China found that depressive symptoms at baseline predicted follow-up PMPU, but PMPU at baseline did not predict follow-up depressive symptoms [38]. In light of these inconsistent findings, it is necessary to conduct a longitudinal study to further explore the association.

### Sleep quality

Sleep may be a major influencing factor for PMPU and depressive symptoms, because studies have indirectly shown that there may be biological and psychological connections among the three factors. A German study found adolescents with sleep disorders more likely to use smartphones longer because they used smartphones as a coping mechanism to suppress worries [39]. This indirectly indicated that poor sleep quality may cause individuals to develop PMPU. Studies have shown that in individuals with PMPU, constant exposure to blue light can inhibit the secretion of melatonin, and cause sleep and circadian rhythm disorders [40, 41], which might be an important factor in the generation of psychopathological symptoms such as depression [42]. Previous studies have explored the mediating role of sleep quality between PMPU and depressive symptoms using cross-sectional study designs [43, 44]. However, there have also been studies with conflicting results, in which depressive symptoms played a mediating role between PMPU and sleep quality [45]. Moreover, considering the limitations of cross-sectional research, these studies have not confirmed the direction of effects among these factors. Fortunately, previous longitudinal studies have found bidirectional relationships between PMPU and sleep quality [38]; that is, sleep quality predicted PMPU and vice versa. In addition, longitudinal studies show that insufficient sleep and suboptimal sleep quality can predict subsequent depressive symptoms [46, 47]. However, these studies also have certain limitations in that they focused on the longitudinal relationship between the two, rather than explored the relationship among the three. Given that the literature on the relationship between PMPU, sleep quality, and depressive symptoms and its direction is still inconclusive and incomplete, more longitudinal studies on the relationship among the three are warranted.

### Bedtime procrastination

In addition to sleep quality, there may be other sleep factors associated with PMPU and depressive symptoms. The basic characteristics of PMPU are mobile device overuse and lack of self-control. According to the Displacement Hypothesis of The Internet [48], everyone’s time is constant. The more time and energy an individual spends using a mobile phone, the less time and energy they spend on other activities and tasks (such as sleep), which leads to the delayed completion of those activities and tasks. Moreover, according to the Strength Model of Self-Control [49], individuals with PMPU need to access limited and domain-general psychological resources in order to develop self-control. Such resources include the ability to suppress their impulse to use their mobile phone, interpersonal communication, emotion regulation, and judgment and decision-making around online activities. The theory of self-regulation failure suggested that procrastination is the result of the exhaustion of self-control resources and the failure of self-regulation [50]. These theories hint that PMPU may be closely related to individual procrastination, which has been confirmed empirically in cross-sectional studies of nursing students [51]. Recently, special procrastination behavior related to sleep has attracted the attention of researchers. Bedtime procrastination refers to the situation where individuals deliberately delay going to bed or refuse to do so without external interference [52]. Previous research has reported that bedtime procrastination played a mediating effect in the positive correlation between PMPU and sleep quality in Chinese college students [53]. In addition, studies have also found that trait procrastination and general procrastination may lead to PMPU [54, 55]. From personality trait theory we know that personality affects behavior [56]; bedtime procrastination, as a characteristic of the procrastinating personality, may then also affect PMPU, but no research has focused on this possible relationship. Meanwhile, a cross-sectional study in China found that bedtime procrastination was positively associated with depressive symptoms in medical students [57]. Bedtime procrastination means that college students’ sleep time may be reduced, and a previous study has confirmed that short sleep increases the risk of mental disorders in young adults aged 17–25 years [58]. Moreover, a survey of 802 young people with clinically diagnosed depression showed that about 18% had habitual delayed sleep onset [59], and a longitudinal study of adolescents found that individuals with depressive symptoms were more likely to delay bedtime [60]. These studies provide some support for the bidirectional relationship between bedtime procrastination and depressive symptoms. However, further longitudinal studies are needed to confirm this. In addition, according to the Procrastination-Health Model [61], habitual procrastinators will experience pressure caused by missed deadlines or completing tasks at the last moment, and may participate in various unhealthy behaviors (such as using mobile phone before bedtime) that can provide immediate satisfaction. However, stress and unhealthy behaviors have been recognized as important risks for shortened sleep time and decreased sleep quality [62]. A previous cross-sectional study of Chinese college students reported that bedtime procrastination was related to sleep quality [63]. However, to our knowledge, no previous study has longitudinally investigated the association between bedtime procrastination and sleep quality.

In summary, there were known to be close relationships among PMPU, bedtime procrastination, sleep quality, and depressive symptoms. However, the existing studies have mainly conducted simple correlation analyses of two or three out of these four variables or only performed systematic reviews; none systematically included all four variables. Therefore, this study aims to explore the longitudinal relationships among PMPU, bedtime procrastination, sleep quality, and depressive symptoms in Chinese college students.

## Methods

### Participants and procedure

We used the stratified cluster sampling method to select college students from 30 classes at a comprehensive university (including many disciplines and majors, such as science and engineering, humanities and social sciences, medicine) in Shandong Province, China, to conduct a questionnaire survey. We contacted the university administration and with their help obtained a list of all classes. We randomly selected ten classes from among freshmen, sophomores, and juniors with whom to conduct the survey. Seniors, or fourth-year students, were not considered since they would have graduated when the second survey was conducted. The participants’ inclusion criteria were 18 years of age or older, own a mobile phone, no family history of mental disorders (such as depression or anxiety) and no clinically diagnosed affective, substance dependence, or addictive disorders (based on self-report). In December 2019 (T1), 1235 students participated in the baseline study. After 12 months (T2), 1181 participants (582 males and 599 females; age: Mean = 18.91 years, SD = 0.85) completed the questionnaire again, and 54 participants (19 males and 35 females; age range was 18–21 years old, with an average age of 18.47, SD 0.52 at T1) were omitted due to absence from school. All surveys were completed in the classroom, where well-trained investigators guided participants to complete the written questionnaires within 30 min. It should be noted that the investigators’ guidance did not involve discussion of the content of the questionnaire, but only filling instructions, such as indicating where to fill in the answers and keeping time for the test-takers. Before the survey, all students signed an informed consent form. This study was approved by the Medical Ethics Committee of the Second Affiliated Hospital of the Shandong University of Traditional Chinese Medicine, and all participants provided written informed consent. The Chinese Civil Law stipulates that 18-year-old citizens have full capacity to conduct civil activities according to their own actions and willingness. Therefore, the participants in the survey did not need the consent of their parents or caregivers.

### Measurements

#### MPATS

PMPU was measured using the MPATS, developed by Xiong et al. [27] and widely used to evaluate the PMPU of college students, showing good reliability and validity in China [64]. This scale includes 16 items with a four-factor structure, including withdrawal symptoms (six items), salience (four items), social comfort (three items), and mood changes (three items). All items are rated on a 5-point Likert-type scale ranging from 1 (very inconsistent) to 5 (very consistent). The total score is the sum of the scores of the four factors, which ranges from 16 to 80; the higher the total score, the greater the level of PMPU. The Cronbach’s alpha was 0.91 and 0.94 at T1 and T2, respectively. Further, to ensure that the PMPU we measured is consistent with the concept proposed by this study, we shared a guideline before administering the MPATS—mobile phone use in this scale refers to use that is not for purpose of self-improvement, increase productivity or search for social support (such as work, study, and communication with families and friends), but for these with the motivation and purpose of relieving negative emotions, relaxing oneself, and satisfying online social and entertainment needs.

#### The bedtime procrastination scale (BPS)

Bedtime procrastination was measured using the BPS [65], which was translated into Chinese and has been used in previous studies [57]. There are nine items in this scale. A sample item is “I do not go to bed on time,” rated on a 5-point Likert scale, with responses ranging from 1 = never to 5 = always. Higher total scores reflect increased degrees of bedtime procrastination. The Cronbach’s alpha of the scale was 0.80 and 0.79 at T1 and T2, respectively.

#### The Pittsburgh sleep quality index (PSQI)

Sleep quality was measured using the PSQI [66], which includes 19 items divided into seven dimensions: subjective sleep quality, sleep latency, sleep duration, sleep efficiency, sleep disturbance, use of sleeping medication, and daytime dysfunction. A Likert 4-level scoring method (0–3 points) was used for each dimension, for a total score of 0–21 points; the higher the score, the lower the sleep quality of the subjects. We used the Chinese version of the PSQI in this study [67]. The Cronbach’s alpha of the scale was 0.70 and 0.73 at T1 and T2, respectively.

#### The patient Health Questionnaire-9 (PHQ-9)

The PHQ-9 was used to evaluate the frequency of depressive symptoms over the past two weeks [68]. This scale had been translated into Chinese, and showed good psychometric properties in the general population [69]. It is composed of nine items, with 0, 1, 2, and 3 points corresponding to “no,” “several days,” “more than half of the days,” and “almost every day,” respectively, for a total score of 0–27 points; the higher the score, the more serious the depressive symptoms. The Cronbach’s alpha of the scale was 0.86 and 0.89 at T1 and T2, respectively.

### Data analysis

The descriptive statistics and Pearson’s correlation analysis were conducted using SPSS version 25.0 (IBM Corporation, Armonk NY, USA) for Windows. Data are presented as n (%) for categorical variables and mean ± SD for numerical variables. We used AMOS 23.0 software to perform cross-lagged panel analysis. First, we evaluated the longitudinal measurement invariance of the four scales used in this study, including configural invariance, metric invariance, and scalar invariance [70]. Second, adjusting gender and age, we constructed a cross-lagged model to test the longitudinal bidirectional relationships among PMPU, bedtime procrastination, sleep quality and depressive symptoms in college students. Model fit was evaluated using comparative fit index (CFI), Tucker–Lewis index (TLI), root–mean–square error of approximation (RMSEA) and standard root–mean–square (SRMR). According to previous studies [71], CFI and TLI greater than 0.90 and RMSEA and SRMR less than 0.08 indicate that the model fit is acceptable. Considering that chi-squared is sensitive to sample size, we did not use chi-squared as an indicator of model fit [72]. Meanwhile, we calculated 95% confidence intervals (CI) using a bias-corrected bootstrap sample that was repeated 5000 times. The 95% CI did not include zero, indicating that the effect was statistically significant (*p*-value < 0.05). In addition, we used the change values of CFI (ΔCFI) and RMSEA (ΔRMSEA) to evaluate the measurement invariance. When ΔCFI≤0.01 and ΔRMSEA≤0.015, the measurement invariance model was acceptable [73].

## Results

### Measurement invariance test

In order to test the longitudinal measurement invariance of the scales, we first established configural invariance models. The results showed that the configural invariance models of the four scales all fitted well. Subsequently, we set the factor loadings to be equal over time and established metric invariance models. All model fits were good. The fit results of the metric invariance models showed that ΔCFI and ΔRMSEA were both less than 0.01, indicating invariance of factor loadings on each scale over time. On the basis of the metric invariance model, we further restricted the equality of thresholds, to test scalar invariance; ΔCFI and ΔRMSEA were still within the acceptable range. These results indicate that the four scales have measurement invariance at two time points. More results about the model-fitting index are shown in Multimedia Appendix 1.

### Descriptive and correlational analyses

Table 1 shows the means, standard deviations, and correlations of the variables. Correlation analysis showed statistically significant correlations among the four variables of PMPU, bedtime procrastination, sleep quality, and depressive symptoms at two time points.

**Table 1:** Descriptive and correlations analyses for variables

Variables	1	2	3	4	5	6	7	8
1. Problematic mobile phone use-T1	–							
2. Bedtime procrastination-T1	0.435*	–						
3. Sleep quality-T1	0.365*	0.442*	–					
4. Depressive symptoms-T1	0.431*	0.362*	0.571*	–				
5. Problematic mobile phone use-T2	0.526*	0.328*	0.277*	0.364*	–			
6. Bedtime procrastination-T2	0.283*	0.458*	0.334*	0.236*	0.340*	–		
7. Sleep quality-T2	0.285*	0.324*	0.494*	0.378*	0.435*	0.417*	–	
8. Depressive symptoms-T2	0.324*	0.301*	0.348*	0.451*	0.496*	0.378*	0.581*	–
Mean	39.20	25.36	4.70	5.76	37.08	24.92	4.02	4.63
Standard deviation	12.27	6.46	2.91	4.37	13.62	5.26	2.93	4.41

*p < .01. T1 = Time 1, T2 = Time 2

### Cross-lagged model

**Fig. 1:**
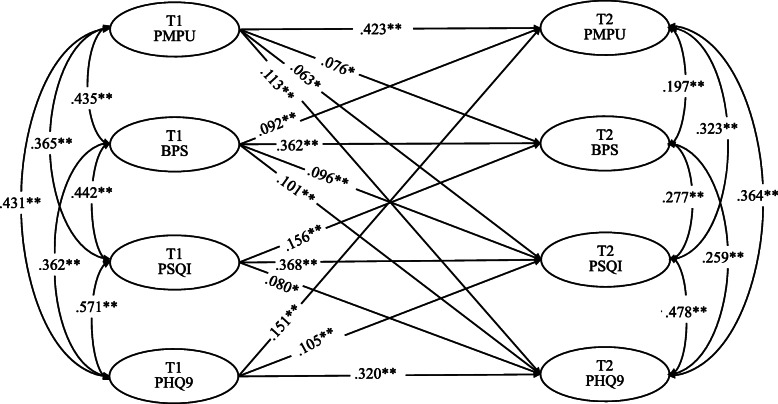
Cross-lagged relationships among PMPU, bedtime procrastination, sleep quality, and depressive symptoms. *Notes:* *p < 0.05, **p < 0.01. Only statistically significant paths are displayed

The cross-lagged model showed good fit to the data (χ^2^(*df*) = 6.682(3), *p* > 0.05; CFI = 0.999, TLI = 0.986, RMSEA = 0.032, SRMR = 0.010). Figure 1 and Table 2 display the results for the cross-lagged model. The results suggest that PMPU at T1 positively predicted bedtime procrastination, depressive symptoms at T2 and vice versa, while sleep quality was only significantly predicted one-way. Bedtime procrastination at T1 positively predicted sleep quality at T2, and vice versa and only significantly positively predicted depressive symptoms one-way. Moreover, sleep quality at T1 positively predicted depressive symptoms at T2 and vice versa.

**Table 2:** Cross-Lagged Model

Path	Effect	SE	LLCI	ULCI
PMPU-T1 to PMPU-T2	0.423	0.031	0.361	0.481
PMPU-T1 to Bedtime procrastination-T2	0.076	0.034	0.008	0.141
PMPU-T1 to Sleep quality-T2	0.063	0.032	0.001	0.126
PMPU-T1 to Depressive symptoms-T2	0.113	0.038	0.037	0.187
Bedtime procrastination-T1 to Bedtime procrastination-T2	0.362	0.035	0.294	0.433
Bedtime procrastination-T1 to PMPU-T2	0.092	0.031	0.032	0.153
Bedtime procrastination-T1 to Sleep quality-T2	0.096	0.029	0.038	0.154
Bedtime procrastination-T1 to Depressive symptoms-T2	0.101	0.031	0.042	0.163
Sleep quality-T1 to Sleep quality-T2	0.368	0.034	0.301	0.435
Sleep quality-T1 to Bedtime procrastination-T2	0.156	0.036	0.087	0.230
Sleep quality-T1 to Depressive symptoms-T2	0.080	0.039	0.002	0.157
Depressive symptoms-T1 to Depressive symptoms-T2	0.320	0.039	0.242	0.398
Depressive symptoms -T1 to PMPU-T2	0.151	0.035	0.083	0.217
Depressive symptoms -T1 to Sleep quality-T2	0.105	0.036	0.033	0.177

*SE* Standard error, *LLCI* lower limit confidence interval 95%, *ULCI* upper limit confidence interval 95%

## Discussion

In this study, we adopted a two-wave longitudinal design and constructed a cross-lagged model to analyze the bidirectional associations among PMPU, bedtime procrastination, sleep quality, and depressive symptoms. These findings help to understand the longitudinal associations and direction of effects among these factors. The results suggest some longitudinal associations in both directions: (1) PMPU with bedtime procrastination and depressive symptoms; (2) sleep quality with bedtime procrastination and depressive symptoms. As far as we know, this is the first study in which bedtime procrastination was bidirectionally associated with problematic phone use and sleep quality. There were also some one-way associations: (1) PMPU predicted sleep quality, and (2) bedtime procrastination predicted depressive symptoms.

This study showed that depressive symptoms were bidirectionally associated with PMPU and sleep quality, consistent with previous studies [37, 46, 74]. However, a recent 6-month longitudinal study in China found that depressive symptoms at baseline predicted follow-up PMPU, as opposed to the converse [13]. A possible reason was that the length of follow-up was different, which may lead to different directions of effect between PMPU and depressive symptoms. Two previous longitudinal studies, of more than 1 year each, in South Korea found a bidirectional relationship between PMPU and depressive symptoms [37, 75]. However, a 3-month short-term longitudinal study in the United States found only a one-way association, in which PMPU at baseline predicted follow-up depressive symptoms [76]. This may mean that it will take longer to observe the bidirectional relationship between the two. However, there have also been longer than 1 year longitudinal studies that found only a one-way relationship [38, 77]. Therefore, it is necessary to conduct further longitudinal studies, with longer follow-up periods, to discover the long-term association between PMPU and depressive symptoms. Above all, our findings suggest that the formulation of effective interventions to reduce PMPU and depressive symptoms is an important topic that needs to be addressed urgently.

In addition, the relationship between sleep problems and depressive symptoms has also been widely confirmed. Breslau et al. pointed out that sleep disturbance was an important predictor of depression in young adults [78]. Moreover, many studies have found biological links between the two factors. With regard to inflammatory biomarkers, sleep disorders may lead to increased levels of CPR and IL-6, and inflammatory reaction might be part of the pathophysiological process of depression [79]. From the perspective of neurobiology, sleep/circadian rhythm is related to dopaminergic and serotonergic functions [80], and these neurotransmitters play an important role in emotion regulation and the pathogenesis of depression. In addition, some studies have found that sleep quality plays a mediating role between PMPU and physical symptoms [81]. All of these findings support the view that sleep quality is closely associated with depressive symptoms.

This study also found that PMPU could only predict sleep quality in one direction, consistent with the results of previous longitudinal study [82]. Previous studies have found that PMPU was associated with a variety of sleep problems. For example, a three-year longitudinal study of Korean children and youth found that PMPU was associated with sleep quality [82], and a cross-sectional study of Japanese high school students showed that using mobile phones for more than five hours a day was associated with short sleep duration and insomnia [83]. It is noted that our research has not found that T1 sleep quality could predict T2 PMPU, different from a previous study finding a bidirectional association [38]. Previous studies showed that poor night sleep quality was correlated with daytime tiredness and sleepiness [84, 85]. It could mean that college students with poor night sleep quality may experience daytime fatigue and sleepiness, which results in not much time and energy to use mobile phones, reducing the frequency and time of mobile phone use, thereby decreasing the probability of PMPU.

In this study, bedtime procrastination was bidirectionally associated with PMPU and sleep quality. This finding also supports Sirois’s view that procrastination increases individuals’ health risks [86]. Although this has not been reported in the literature, a cross-sectional study used a mediation model to preliminarily analyze the directions of the effect: PMPU indirectly affects sleep quality by affecting bedtime procrastination [53]. According to Steel’s Temporary Motivation Theory (TMT) [87], whether an individual exhibits procrastination depends on the perceived utility of the task to that individual, and this comprehensive variable is related to value, expectation, and delay. For individuals with PMPU, the time needed to obtain the health benefits of sleep was far longer than the time spent experiencing the immediate psychological satisfaction and pleasure obtained through mobile phone use. Thus, the former was far less attractive to individuals than the latter, which explains why mobile phone addicts tend to defer sleep tasks in exchange for relatively high perceived utility, resulting in bedtime procrastination. This also supports the Displacement Hypothesis of The Internet to a certain extent; that is, PMPU consumed the limited time of individuals and delayed their bedtime. On the other hand, when bedtime procrastination becomes a habit, it means that individuals have more time and opportunities to engage in other activities. For example, mobile phone use may be the primary choice for bedtime procrastinators, because the mobile phone’s strong accessibility and powerful functions [76] could achieve their immediate happiness and satisfaction, which increases the probability of PMPU. In addition, a longitudinal study of 633 middle school students also showed that academic procrastination is an antecedent to PMPU [88]. These findings indicate that PMPU and bedtime procrastination may have a mutual causal relationship and form a vicious circle.

The same was true for the association between bedtime procrastination and sleep quality. Bedtime procrastination might lead to disordered sleep and the reduction of subjective sleep time. At the same time, procrastinators need to bear the pressure of going to bed at the last moment and falling asleep quickly, which leads to a decline in sleep quality. In addition, neurophysiological studies have shown that poor sleep quality can disrupt the natural circulation of different sleep stages [89, 90], thereby affecting the activation and inactivation of the prefrontal cortex (PFC) of the brain [91–93], resulting in a decrease in the executive function [94]. It is needed to note that self-regulation is heavily dependent on activation levels of PFC [95], and previous studies have found that decreased executive function can lead to self-regulation failure through various ways such as resource depletion and temporary reduction of blood sugar [96]. In other words, poor sleep quality may reduce the activity of the prefrontal cortex of the brain, resulting in self-regulation failure. Previous studies have also found that poor sleep quality can consume an individual’s energy and self-regulation resources and predict failure of individual self-regulation at work [97], thereby resulting in work procrastination [98]. This may explain why T1 sleep quality can predict T2 bedtime procrastination, since procrastination was considered to be the result of the failure and reduced resources of self-regulation according to the self-regulation view of procrastination [99, 100]. Therefore, individuals with poor sleep quality may experience self-regulation failure (e.g., inability to resist temptation) [98], making them easily attracted to other things or activities before going to sleep, and consequently leading to bedtime procrastination.

Furthermore, we found that bedtime procrastination at T1 predicted depressive symptoms at T2. A previous longitudinal study in Japan found a significant bidirectional relationship between adolescents’ bedtime procrastination and the following year’s depression/anxiety [60]. However, the opposite path, from depressive symptoms to bedtime procrastination, was not statistically significant. This may be related to the difference between the bedtime procrastination assessment tools used in the two studies. Previous studies mainly used self-reported bedtime differences to reflect bedtime delay [60], while this study used a specialized scale. Therefore, the longitudinal association between bedtime procrastination and depressive symptoms needs further exploration. Based on the one-way path from bedtime procrastination to depressive symptoms, procrastination was characterized by the priority of current self over future self and the priority of short-term emotion regulation over long-term goals and rewards [101]. When individuals with bedtime procrastination realize that they do not go to bed on time at the expense of their health, self-imposed pressure will follow, thereby causing depressive symptoms. Previous studies suggest that procrastination was associated with guilt, shame, and negative self-evaluation [101]; these adverse psychological factors may eventually lead to the occurrence of depressive symptoms. In addition, bedtime procrastination may also indirectly affect depressive symptoms through sleep quality; that is, bedtime procrastination reduces the individual’s sleep quality, and poorer sleep quality causes the individual to develop depressive symptoms. This means that sleep quality may have a mediating effect between bedtime procrastination and depressive symptoms, but this requires further multi-wave longitudinal studies for verification.

### Implications and limitations

These findings have certain theoretical and practical significance for reducing PMPU, sleep problems, and depressive symptoms. Theoretically, this study has preliminarily clarified the longitudinal relationships and direction of effects among PMPU, bedtime procrastination, sleep quality, and depressive symptoms. Previous research has focused on studying the cross-sectional correlations between these variables and explored the mediating effect of sleep variables between PMPU and depressive symptoms. However, due to the unclear direction of the effect, these mediation studies cannot demonstrate causality, and many of the research results are contradictory. Moreover, while a few longitudinal studies have explored the association between two of these variables, they did not incorporate multiple variables to explore their comprehensive relationships. These studies offered the foundation for the next step in the study of the association mechanism. Practically speaking, this study provides a new intervention strategy from the perspective of bedtime procrastination and sleep quality to reduce PMPU and depressive symptoms. Interventions for bedtime procrastination may be suitable for efforts to mitigate PMPU, sleep quality, and mental health problems based on the research findings that bedtime procrastination may be the antecedent for PMPU, sleep quality, and depressive symptoms. We propose the following suggestions for interventions. First, technology-based interventions are necessary. For example, mobile phone software developers can create programs to provide users with regular reminders, forced dormancy, or exit from running apps, to reduce mobile phone use. Meanwhile, the program can record the daily usage, duration, and unlock times of the phone and feed them back to the user the next day. What needs special attention is clarifying the motivation and reason behind PMPU, which is the key to prompt users to change. How to integrate psychological elements and accurately identify the motivation of different users to provide personalized digital intervention schemes should be a major direction for software developers in the future [102]. Second, university educators should publicize the potential hazards of PMPU, sleep problems, and depression symptoms as well as present relevant coping strategies, such as daily setting of small goals for reducing mobile phone use and sleeping on time; immediate regulation of negative emotions and self-control training through class meetings, speeches, brochures, and other relevant practices may also be beneficial. Third, organizing a scientific bulletin with sleep hygiene in dormitories and establishing mutual aid groups in dormitories (e.g., dormitory members reminding each other to sleep on time) can help students reduce the use of mobile phones at night and develop a habit of going to bed on time.

There are some limitations to our research. First, this longitudinal study only had two waves, which cannot clarify the underlying mechanisms among PMPU, bedtime procrastination, sleep quality, and depressive symptoms (for example, whether sleep procrastination and sleep quality will mediate the relationship between PMPU and depressive symptoms, or whether depressive symptoms will mediate the relationship between PMPU and sleep quality). Multi-wave (three or more measuring points) studies can help resolve this limitation. Moreover, since the study used a non-experimental design, our research may be unable to determine causal relationships among these four variables; in future, intervention studies will be needed to infer the cause and effect. In addition, the data were collected using self-reporting methods, which may lead to information bias. Additionally, all participants were selected from one university in Shandong Province, so caution should be exercised when extrapolating the research results within China or especially to other countries. Finally, since we only paid attention to the directions and paths of influence among the aforementioned four variables, the study offers limited insights into those specific theories that can explain the relationship among them. In the future, it is necessary to adopt experimental designs to determine various factors related to these theories in a holistic way in order to eliminate or control potential confounding and obtain robust and repeatable results to further support, oppose, or verify these theories.

## Conclusion

This study further expands our understanding of the longitudinal and bidirectional relationship among PMPU, bedtime procrastination, sleep quality, and depressive symptoms. In addition, it will assist school mental health educators in designing targeted interventions to reduce PMPU, sleep problems, and depressive symptoms among college students.

## Supplementary Information

**Additional file 1: Multimedia Appendix 1**. Model fit statistics for tests of measurement invariance.

## Data Availability

Data can be obtained by contacting the corresponding author with appropriate reasons.

## Abbreviations

PMPU: Problematic mobile phone use
CFI: Comparative fit index
TLI: Tucker–Lewis index
RMSEA: Root–mean–square error of approximation
SRMR: Standardized root–mean–square residual
