# Supplementary material for: Longitudinal relationships among problematic mobile phone use, bedtime procrastination, sleep quality and depressive symptoms in Chinese college students: a cross-lagged panel analysis
Source: BMC Psychiatry. 2021 Sep 10;21:449. doi: 10.1186/s12888-021-03451-4 (PMC8431882; doi:10.1186/s12888-021-03451-4)
Supplement: Supplementary file 1 — Additional file 1: Multimedia Appendix 1. Model fit statistics for tests of measurement invariance. [file 12888_2021_3451_MOESM1_ESM.docx]

**Multimedia Appendix 1**

**Model fit statistics for tests of measurement invariance**

|  | Problematic mobile phone use | | | Bedtime procrastination | | | Sleep quality | | | Depressive symptoms | | |
| --- | --- | --- | --- | --- | --- | --- | --- | --- | --- | --- | --- | --- |
| Variables | Configural Invariance | Metric Invariance | Scalar Invariance | Configural Invariance | Metric Invariance | Scalar Invariance | Configural Invariance | Metric Invariance | Scalar Invariance | Configural Invariance | Metric Invariance | Scalar Invariance |
| χ2 | 1095.40 | 1159.95 | 1293.52 | 275.13 | 339.91 | 400.11 | 133.56 | 157.427 | 199.71 | 271.55 | 318.65 | 355.061 |
| *df* | 349 | 365 | 381 | 100 | 108 | 116 | 52 | 58 | 65 | 93 | 101 | 109 |
| CFI | 0.964 | 0.961 | 0.956 | 0.975 | 0.967 | 0.957 | 0.978 | 0..973 | 0.965 | 0.981 | 0.976 | 0.972 |
| TLI | 0.949 | 0.947 | 0.942 | 0.962 | 0.952 | 0.932 | 0.961 | 0.959 | 0.946 | 0.968 | 0.964 | 0.959 |
| RMSEA | 0.042 | 0.043 | 0.045 | 0.039 | 0.043 | 0.051 | 0.036 | 0.038 | 0.043 | 0.040 | 0.043 | 0.046 |
| SRMR | 0.033 | 0.037 | 0.043 | 0.033 | 0.048 | 0.050 | 0.026 | 0.030 | 0.035 | 0.026 | 0.032 | 0.041 |
| △CFI |  | 0.003 | 0.005 |  | 0.008 | 0.010 |  | 0.005 | 0.008 |  | 0.005 | 0.004 |
| △RMSEA |  | 0.001 | 0.002 |  | 0.004 | 0.009 |  | 0.002 | 0.005 |  | 0.003 | 0.003 |
